# ShaPINg Cell Fate Upon DNA Damage: Role of Pin1 Isomerase in DNA Damage-Induced Cell Death and Repair

**DOI:** 10.3389/fonc.2014.00148

**Published:** 2014-06-16

**Authors:** Tilman Polonio-Vallon, Daniel Krüger, Thomas G. Hofmann

**Affiliations:** ^1^Research Group Cellular Senescence, German Cancer Research Center (DKFZ), DKFZ-ZMBH Alliance, Heidelberg, Germany

**Keywords:** pin1, apoptosis, DNA repair, p53, p73, HIPK2, CtIP

## Abstract

The peptidyl–prolyl *cis/trans* isomerase Pin1 acts as a molecular timer in proline-directed Ser/Thr kinase signaling and shapes cellular responses based on recognition of phosphorylation marks and implementing conformational changes in its substrates. Accordingly, Pin1 has been linked to numerous phosphorylation-controlled signaling pathways and cellular processes such as cell cycle progression, proliferation, and differentiation. In addition, Pin1 plays a pivotal role in DNA damage-triggered cell fate decisions. Whereas moderate DNA damage is balanced by DNA repair, cells confronted with massive genotoxic stress are eliminated by the induction of programed cell death or cellular senescence. In this review, we summarize and discuss the current knowledge on how Pin1 specifies cell fate through regulating key players of the apoptotic and the repair branch of the DNA-damage response.

## Introduction

Proline residues bear the unique intrinsic feature of being able to convert between two distinct conformational states in a protein. Since the dihedral angle (ω) of the proline bond is large, a switch can introduce significant changes in total protein structure (1, 2). The interconversion between the two isomeric *cis* and *trans* states of peptide bonds preceding the amino acid proline can be catalyzed by peptidyl–prolyl *cis/trans* isomerases (PPIases) (3).

Three distinct families of PPIases that can facilitate prolyl isomerization have been identified so far. Among those are the family of Cyclophilins (Cyp), FK506-binding proteins (FKBPs), and the parvulins (4–7). Both Cyp and FKBPs have strong implications in immune responses due to their function as receptors for the immunosuppressive drugs cyclosporin A and FK506, respectively (4, 8, 9). The family of parvulins consists of the PPIase NIMA-interacting 1 (Pin1) and the more distantly related subgroup of proteins Par14 (Pin4) and Par17, which are both encoded within a single locus in the human genome (10, 11). In contrast to Pin1, the biological functions of Par14 and Par17 remain currently largely obscure. In the following section, we will introduce Pin1, which shows a unique feature among the PPIase protein family members, as it recognizes its client proteins in a phosphorylation-specific manner.

## Phospho-Specific Isomerase Pin1

**Figure 1:**
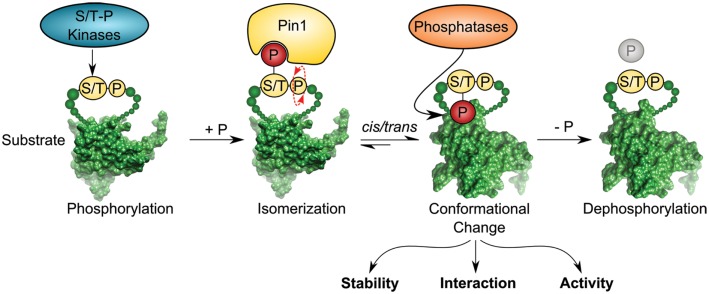
**Pin1 isomerase induces *cis/trans* conformational change of substrates containing pSer/Thr–Pro motifs**. Ser/Thr–Pro-directed kinases phosphorylate diverse substrates thereby creating a putative binding site for the foldase Pin1, which catalyzes *cis/trans* isomerization of the previously phosphorylated protein in a subsequent step. Isomerization can regulate different functions of the substrate, such as stability, interaction, and activity. Pro-directed phosphatases, such as PP2A can dephosphorylate the pSer/Thr–Pro isomer [depicted substrate has been downloaded from PDB database; Protein-ID: 2FEJ (111)].

Pin1 is a small enzyme consisting of 163 amino acids. It contains a WW protein interaction domain, which recognizes short proline-rich motifs at its N-terminus, and a C-terminal PPIase domain. The enzymatic conversion of peptide bonds between *cis* and *trans* conformation is dependent on the phosphorylation state of the Ser/Thr–Pro motif, which is the target sequence of Pin1 (12–14). In contrast to other known PPIases, Pin1 has the unique property of recognizing phosphorylation-specific motifs for isomerization. This feature links occurrence of specific phosphorylation-marks sites to conformational changes of its client proteins by *cis/trans* isomerization of the phospho-Ser/Thr–Pro bond (15) (Figure 1). Ser/Thr phosphorylation is a key mechanism of signal transduction and the most frequent post-translational modification in the cell. Phosphorylation at serine and threonine residues accounts for around 96% of all protein phosphorylation in the cell as revealed by global mass spectrometry analysis (16). Although phosphorylation has been shown to be sufficient for inducing conformational changes *per se* (17, 18), Pin1-catalyzed isomerization of phospho-Serine/Threonine residues represents a central mechanism in signaling and acts as a trigger to alter protein conformation (19–23).

Many Ser/Thr–Pro-directed kinases are predominantly localized in the nucleus (24) and play a major role in cell cycle regulation and cellular stress responses. This is evident from the well-studied functions of some representatives of this subgroup of kinases, such as cyclin-dependent kinases (CDKs), Jun-N-terminal protein kinases (JNKs), polo-like kinases (PLKs), and glycogen synthase kinase 3 (GSK-3). Accordingly, Pin1 also predominantly localizes to the nucleus, where it exerts its versatile signaling functions in regulating mitosis and mediating stress responses (14, 15, 25).

In this review, we are going to focus on the role of Pin1 in DNA-damage signaling. Excellent comprehensive reviews covering the function of Pin1 in mitosis, Alzheimer disease, immune response, proliferation control, and cancer biology have been published recently (19, 22, 26–31) and are recommended to those readers interested in obtaining a global view on Pin1 function. In the following sections, we attempt to summarize the current knowledge about the function of Pin1 in DNA damage-induced cell fate with a focus on its role in the cell death response and DNA repair.

## Pin1 and p53

**Figure 2:**
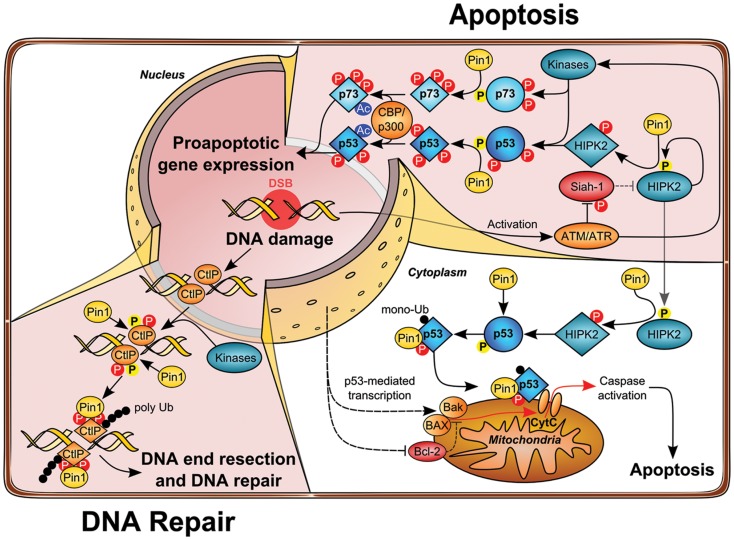
**Role of Pin1 in DNA damage-induced apoptosis and DNA repair pathways**. Upon DNA damage ATM loosens the HIPK2–Siah1 complex by phosphorylation of Siah1, thereby allowing HIPK2 autophosphorylation. Phosphorylated HIPK2 recruits Pin1, which in turn changes HIPK2 conformation and potentiates disruption of the HIPK2–Siah1 complex. HIPK2 gets stabilized and phosphorylates p53 Ser46 to induce apoptosis. Moreover, p53 and Pin1 form a complex which further unleashes p53 apoptotic functions by changing p53 conformation and facilitating acetylation of p53 by the acetyltransferase CBP and loading of p53 on pro-apoptotic target gene promotors. At the same time, prolyl-isomerization of p53 Ser46–Pro47 by Pin1 leads to increased monoubiquitination of p53, which in turn triggers the translocation of cytosolic p53 to mitochondria, thereby initiating mitochondrial outer membrane permeabilization (MOMP) and intrinsic apoptosis release of cytochrome *c* into the cytoplasm. In a parallel signaling branch, ATM activates other kinases, such as c-Abl and p38, which leads to phosphorylation of p73. Recruitment and Pin1 binding leads to association with the acetyltransferase p300 and stimulates acetylation, which enhances transcriptional activity of p73 toward apoptotic genes. Beyond its function in apoptosis, Pin1 plays also a crucial role in double-strand break (DSB) repair. During S/G2 phase CtIP promotes end resection of DNA lesions. Phosphorylation of CtIP by CDK2 and most likely other kinases leads to Pin1 binding and isomerization of CtIP, which promotes its ubiquitylation and proteasomal degradation, thereby counteracting DSB end resection in favor of NHEJ.

The tumor suppressor *p53* is mutated in more than 50% of human cancer (32) and cancer cells expressing wild-type p53 commonly functionally inactivate p53 by other means including overexpression of the p53-degrading E3 ubiquitin ligases MDM2 (33). In response to genotoxic stress such as UV, ionizing irradiation (IR), or chemotherapeutic drug treatment, p53 is stabilized, activated, and drives transcription of target genes leading to cell cycle arrest, senescence, or apoptosis, mostly depending on the strength of the insult and the cellular background (34). Activation of p53 upon genotoxic stress is largely determined by post-translational modifications, including site-specific phosphorylation and acetylation. Notably, p53 is phosphorylated by a set of stress-activated, proline-directed protein kinases such as p38, homeodomain-interacting protein kinase-2 (HIPK2), and DYRK2 (35–39). Pin1 binds to phosphorylated Ser/Thr–Pro sites on p53 upon genotoxic stress. In particular, phosphorylation at Ser33, Ser46, Thr81, and Ser315 has been shown to mediate the interaction of p53 and Pin1 (40–42). Subsequent conformational changes driven by Pin1 are crucial for the functional activation and stabilization of p53 upon DNA damage, which is achieved, at least in part, due to impaired interaction with the E3 ubiquitin ligase MDM2. Since MDM2 is a direct target gene of p53 (43), the Pin1-mediated accumulation of p53 is additionally regulated by a transcription-dependent increase of MDM2 (41). In line with these observations, cell cycle checkpoint function is impaired in Pin1-deficient MEFs as shown by higher re-entry into S-phase upon DNA damage. Overall, these studies demonstrate that Pin1 is important for the timely accumulation and the functional activation of p53 resulting in cell cycle arrest or apoptosis (Figure 2).

An important activation mark of p53 is the phosphorylation of p53 at Ser20, which is under control of checkpoint kinase 2 (Chk2) (44, 45). Phosphorylation of p53 at Ser20 impairs the interaction of p53 with its E3 ubiquitin ligase MDM2 (46, 47). Mutation of p53 Pro82 that precedes the phosphorylation site at Thr81 results in impairment of DNA damage-induced phosphorylation of p53 at Ser20 (48). Notably, genotoxic stress has also been shown to result in JNK-mediated phosphorylation of p53 at Thr81, which is important for JNK-dependent p53 transcriptional activation and apoptosis (49). Mechanistically, Pro82 mutation results in reduction of the DNA damage-induced interaction of Chk2 and p53. Exogenous expression of Pin1 enhances the interaction of Chk2 and p53 upon DNA damage and Ser20 phosphorylation is strongly impaired in Pin1-deficient MEFs. These findings indicate that Pin1-mediated isomerization of the Thr81–Pro82 bond is important for the binding of Chk2 to p53 and for DNA damage-induced phosphorylation of p53 at Ser20. In conclusion, this mechanism provides a model of how Pin1 facilitates Chk2-mediated p53 phosphorylation at Ser20, and as a functional consequence, leads to the disruption of the p53–MDM2 complex (48, 50).

Since Pin1 has a potent role in the activation of p53, one might wonder whether Pin1 is also able to trigger the activation of mutant p53. Somatic mutations in the *TP53* gene are frequent in many cancer types and have a huge impact on the clinical outcome of those cancers (51, 52). Pin1 is frequently overexpressed in cancer and mediates proliferative signals through client proteins such as Cyclin D1 (53, 54). *TP53* R172H mutation corresponds to the hot-spot mutation 175 in human cancers and has been linked to gain of function mechanisms associated with tumor progression, resembling Li–Fraumeni syndrome. In fact, *TP53* missense mutations exhibit enhanced oncogenic potential beyond the loss of physiological p53 functions (52, 55).

Comparison between mice harboring a mono-allelic mutant *p53*^R172H^ or *p53* KO mice in a *Pin1* wild-type or *Pin1*-deficient background revealed that absence of Pin1 results in a reduced tumor frequency and a decreased incidence of hematopoietic cancers. Most importantly, cancers of epithelial origin were completely absent in *p53*^R172H^; *Pin1* KO mice (56). Interestingly, mutant p53 appears to be constitutively phosphorylated on Ser46 and Ser33 in breast cancer cell lines, which is potentiated upon oncogenic Ras^G12V^ induced signaling, thus creating permanent target sites for Pin1. Furthermore, mutant p53 exhibits a highly metastatic phenotype that is dependent on Pin1, since Pin1-depletion results in strongly reduced migration and invasion capacity *in vitro* and *in vivo*. Inversely, Pin1 overexpression potentiated migration in a mutant p53-dependent manner. Moreover, Ser46 phosphorylation appears to be critical for the migration phenotype observed in breast cancer cell lines bearing mutant p53. Most importantly, both Pin1 and mutant p53 synergize in the positive regulation of a set of genes that are relevant for migration and invasion. Furthermore, the oncogenic functions of mutant p53 are further enhanced by augmenting p63 transcription in a Pin1-dependent fashion, thereby reprograming gene expression in breast cancer cells. Overall survival appears to be drastically diminished in breast cancer cases bearing p53 missense mutation and Pin1 overexpression, which suggests that p53 status in combination with Pin1 expression level can be used as an independent prognostic marker for poor clinical outcome.

## Pin1 and p73

The p73 protein is a member of the p53 protein family. Similar to its homolog p53, p73 harbors tumor suppressive functions such as growth suppression, apoptosis, DNA repair, senescence, and differentiation. DNA-damage induced by chemotherapeutic drugs such as Doxorubicin or Cisplatin also activate p73 (in the absence of p53), which exerts transcription-dependent and independent functions (57–60). Apart from being implicated in cytotoxic stress-mediated cell cycle arrest or apoptosis, p73 also plays a pivotal role in development, especially in that of the neuronal system (61).

Interestingly, it has been demonstrated that DNA damage-induced p53-dependent apoptosis requires functional p63 and p73 (62). Along these lines, Pin1 is not only essential for proper activation of p53, but it is also required for p73’s pro-apoptotic function (63). Pin1-depletion leads to defective induction of apoptosis and p73 accumulation upon Cisplatin administration. Accordingly, Pin1 controls p73 turnover in unstressed cells and upon cytotoxic stress and is required for proper activation of p73 target genes such as Bax and PIG3, as revealed in cell lines lacking p53 expression (63). In contrast to p53, Pin1 interacts with p73 even without any indication of cellular stress, implying that p73 is constitutively phosphorylated at Pin1 binding sites. Nevertheless, treatment with Doxorubicin and Cisplatin enhances binding of Pin1 to p73 via Ser412, Thr442, and Thr482 target sites, which is partly due to activation of p73 by c-Abl and p38 kinase (63). In fact, Pin1 is required for the c-Abl induced p73 activation and accumulation upon cytotoxic stress and p300-mediated p73 acetylation is strongly impaired in Pin1-deficient cells, since Pin1-mediated isomerization regulates p300–p73 binding (63). In conclusion, these data support the indispensable role of Pin1 activity to synergistically drive p53 and p73-mediated apoptosis under given stress conditions (as shown in Figure 2).

## Pin1 and iASPP

p53 activity needs to be tightly regulated in unstressed cells to prevent an unscheduled activation of the apoptotic response. To this end, p53 is sustained at low levels under physiological conditions, which is achieved by constant proteasomal degradation of p53 mediated by the E3 ubiquitin ligase MDM2. On the other hand, also association with iASPP inhibits p53 by preventing its binding to promoters of pro-apoptotic target genes (64). ChIP experiments revealed that under genotoxic stress conditions, Pin1-depletion results in decreased binding of p53 to p21 and Bax promoters. Upon genotoxic stress, Pin1 is directly associated with p53 on chromatin mainly via its binding sites Ser33, Ser46, Thr81, and Ser315. In fact, Pin1 activates p53-mediated transcription in a direct manner by potentiating the binding of the acetyltransferase p300 and subsequent acetylation of p53 at Lys373 and Lys382. Moreover, Pin1 also enhances the binding of p300 on p53-occupied promoters, which results in transcriptional activation. In addition, Thr81–Pro82 also appears to be a critical binding site for Pin1 and is required for proper acetylation of p53. The Thr81 residue lies within the Proline-rich domain of p53, which mediates binding of the p53 inhibitor iASPP. iASPP has been shown to regulate the transcriptional activity of p53 and is also pivotal for the stabilization of p53 in response to genotoxic stress (64). Intriguingly, Pin1 regulates the dissociation of the iASPP–p53 complex upon cytotoxic stress and thereby unmasks p53 transcriptional activity. Notably, dissociation of iASPP from p53 is dependent on stress-induced Ser46 phosphorylation but not on Pin1 stimulated acetylation of p53. Taken together, these findings indicate that Pin1 regulates p53 activity at different levels: (1) Pin1 controls p53 stabilization, (2) Pin1 is required for p53 binding to its target promoters, (3) Pin1 associates with chromatin and promotes p300-mediated acetylation of p53, and (4) Pin1 initiates the dissociation of p53 from its inhibitor iASPP. As described in the next chapter, Pin1 is also essential for stabilization of the p53 Ser46 kinase HIPK2. By facilitating efficient p53 Ser46 phosphorylation, Pin1 may regulate its own complex formation with p53 and drive the apoptotic response.

## Pin1 and the p53 Ser46 Kinase HIPK2

Homeodomain-interacting protein kinase-2 is a central activator of the apoptotic cell death in development and in response to cellular stress and also acts as a tumor suppressor targeted by cellular and viral oncogenes (37, 38, 65–70). HIPK2 triggers apoptosis induction upon various types of DNA damage including UV radiation, ionizing radiation, and chemotherapeutic drug treatment through catalyzing phosphorylation of p53 at Serine 46, a phosphorylation mark, which drives expression of pro-apoptotic target genes (37, 68, 71–73). In addition, HIPK2 also activates the apoptotic response independent of p53 by regulating the JNK pathway and by targeting the anti-apoptotic transcriptional repressor CtBP for degradation (74, 75). In healthy cells and cells recovering from sublethal DNA damage, HIPK2 is kept inactive through proteasome-dependent degradation mediated by the ubiquitin ligases MDM2, WSB1, and Siah-1 (76–79). In the wake of DNA damage, HIPK2 is stabilized by a mechanism involving the checkpoint kinases ATM and ATR, which facilitate dissociation of the HIPK2–Siah-1 complex by phosphorylation of Siah-1 at Ser19 (77).

Interestingly, HIPK2 stabilization upon DNA damage also requires Pin1 activity (80). It has been recognized that HIPK2 autophosphorylates at multiple sites, which influences its kinase activity (81, 82). In particular, autophosphorylation *in trans* at Thr880/Ser882 through an intermolecular mechanism activates its kinase activity and apoptotic function upon genotoxic stress. Thr880/Ser882 autophosphorylation takes place at an early phase of HIPK2 activation and decreases when HIPK2 is fully stabilized. Furthermore, pThr880/pSer882 is followed by Pro residues and thus represent *bona fide* Pin1 binding sites. In fact, Pin1 binds HIPK2 through this phospho-motif and alters the conformation of the autophosphorylated HIPK2 isoform and mediates stabilization by inhibiting its proteasomal degradation. Loss of Pin1 by genetic deletion or RNA interference results in a lack of DNA damage-induced HIPK2 stabilization and apoptosis induction (80) (as shown in Figure 2). Interestingly, HIPK2 autophosphorylation appears to be evolutionary conserved and is also detected on the zebrafish HIPK2 protein, and induction of apoptosis in zebrafish embryos by ionizing radiation (IR) is regulated by the autophosphorylation of HIPK2. Finally, Pin1 is essential for IR-induced cell death in zebrafish embryos and in human cancer cells, highlighting a fundamental role in the DNA damage-triggered apoptotic response (80). Taken together, these findings support a major role of Pin1 in DNA damage-activated apoptosis signaling.

## Pin1 and Huntingtin

It has recently been shown, that mutant huntingtin (mHtt) is able to induce p53-dependent apoptosis via a Pin1-mediated mechanism (83). Activation and accumulation of p53 has been observed in Huntington disease and recapitulated in transgenic mouse models (84). mHtt, which bears an elongated segment of polyglutamine, is able to trigger the DNA-damage response (DDR) and induce the accumulation and phosphorylation of p53 at Ser46 and Ser15 (83, 85). In fact, Ser46 is critical and sufficient for the induction of apoptosis upon mHtt generated cellular stress. Interestingly, the other Pin1 binding sites identified in previous studies (40–42) are dispensable in mHtt-triggered neuronal death. p53 Ser46 is a Ser/Thr–Pro target site for Pin1 and expression of mHtt can stimulate the interaction of p53 and Pin1. As previously mentioned, isomerization of Ser46–Pro47 bond has been demonstrated to be a prerequisite for the dissociation of the inhibitor iASPP from p53 in order to fully activate p53 transcriptional activity (86). In accordance with these previous findings, disruption of the iASPP–p53 complex is also critical in mHtt-mediated p53 activation. Moreover, Pin1 is necessary for the transcriptional activity of p53 and PUMA-mediated apoptosis induction in mHtt-stimulated cells and neurons derived from a mHtt-knock-in mouse model (83). Furthermore, it has been shown that Ser46 phosphorylation upon mHtt expression is synergistically mediated by HIPK2 and PKC δ (Figure 2). These findings provide an unexpected link between neurodegenerative disease and the apoptotic DDR mediated by HIPK2 and Pin1.

## Pin1 and Mitochondrial Apoptosis Signaling

p53 can regulate apoptosis by transcription-dependent and -independent mechanisms. Recent studies have demonstrated that the interplay of the nuclear and cytoplasmic functions of p53 is crucial for shaping its full apoptotic activity (87). The mitochondrial pathway is predominantly activated in response to IR or other types of cytotoxic stress and is therefore exploited in cancer therapy by ionizing-IR or chemotherapeutic drugs such as doxorubicin. Remarkably, p53 has been demonstrated to modulate Bcl-2 family members that regulate apoptosis by controlling mitochondrial permeability (88, 89). Upon cytotoxic stresses, p53 is partially localized at the mitochondrion and has been demonstrated to induce mitochondrial outer membrane permeabilization (MOMP) (90). In fact, the cytosolic apoptotic function of p53 is rapidly induced upon cytotoxic stress and precedes its transcription-dependent functions. Pin1 has also been linked to the regulation of cytosolic functions of p53. In this context, Pin1 potentiates mitochondrial damage induced by p53 and triggers apoptosis by releasing cytochrome *c* from the mitochondria (91). These effects require Pin1–p53 binding, since mutation of target sites on p53 diminished mitochondrial damage and induction of apoptosis when compared to wild-type p53. In addition, Pin1 is important for the efficient translocation of cytosolic p53 to mitochondria upon treatment with the chemotherapeutic drugs Doxorubicin and Etoposide. Mechanistically, Pin1 binds cytosolic fractions of p53 mainly via its target site at Ser46–Pro47. Ser46 is a target site for the protein kinase HIPK2 that has been shown to increase p53-dependent apoptosis (37, 38). Indeed, HIPK2 is able to cooperate with Pin1 to induce mitochondrial apoptosis and increases the fraction of p53 bound to mitochondria, which is dependent on the catalytic activity of the kinase and presence of Pin1 (91). These results show that HIPK2 is not only critical for the nuclear, transcription-dependent function of p53 but – in cooperation with Pin1 – also regulates activation of p53 at mitochondria.

## Pin1 and Telomeres

Telomeres are chromatin structures capping the ends of chromosomes in order to shield the free DNA ends from degradation and damage. Erosion of telomeres below a critical length has been linked to chromosome end fusion and premature aging (92–95). Dysfunctional telomeres are considered as a permanent source of DNA damage leading to the activation of p53 (96). To avoid detection of chromosome ends as aberrant DNA structures, telomeres are organized in a higher order duplex lariat structure, the T-loop, and sequestered by a specialized macromolecular shelterin protein complex. The telomeric repeat binding factor 1 (TRF1) is part of the shelterin complex and is essential for telomere function. Pin1 has been shown to regulate TRF1 function on telomeres by directly binding to TRF1 through the phospho-Thr149–Pro150 motif (97), which is phosphorylated by CDKs during mitosis. Furthermore, Pin1 negatively regulates TRF1 stability, which requires its PPIase activity and leads to increased binding of TRF1 to telomeres (97). Strikingly, TRF1 stability is increased not only upon Pin1-depletion in human cells, but also *in vivo* as determined in the Pin1 KO mouse model. TRF1 has previously been shown to regulate telomere length (98, 99). Remarkably, Pin1 inhibition leads to progressive telomere shortening through a TRF1-dependent mechanism in human cells and in splenocytes derived from Pin1 KO mice (97). Accordingly, Pin1 nullizygous animals show premature aging phenotypes such as reduced bone radio-density and thinner dermal and epidermal layers along with further hallmarks of accelerated aging (54, 100, 101). Of note, the widely used telomerase-deficient mouse models, which are frequently used to study aging by telomere-erosion require five to six generations to develop efficient telomere shortening and premature aging phenotypes. The fact that Pin1 KO mice show massive telomere erosion and a severe aging phenotype even in the first generation suggests that active resection takes place at the telomere in the absence of Pin1. The molecular basis of this interesting phenotype still remains to be elucidated.

## Role of Pin1 in DNA Double-Strand Repair: The Pin1–CtIP Link

DNA damage is one of the major factors driving genomic instability and carcinogenesis in multicellular organisms. Numerous factors lead to the activation of the DDR, including ionizing radiation, chemotherapeutic drug treatment, and strong hyperproliferative signals as induced by oncogene expression (102). Proper cellular response to DNA damage is needed to suppress carcinogenesis, and this involves a set of gene products that elicit cell cycle arrest, DNA repair, premature senescence, or apoptosis, in accordance with the damage inflicted. The maintenance of genome integrity is accomplished by an elaborate signaling network termed DDR. A major mechanism underlying the cellular response to DNA damage is protein-phosphorylation (103). Accordingly, the master checkpoint kinases ATM, ATR, and DNA-PK play a central role in coordination of the DDR. Double-strand breaks (DSBs) are considered to be a particularly deleterious form of DNA lesion that needs to be repaired by the cell to facilitate cell survival and proliferation.

Two main pathways orchestrate the DNA repair process of DSBs, namely homologous recombination (HR) and non-homologous end-joining (NHEJ). HR represents a highly accurate, error-free repair mechanism, whereas NHEJ is more error-prone. In mammalian cells, DSBs produced for instance by IR are mostly repaired by NHEJ, however, when DNA replication is impeded this leads to stalled replication fork collapses and resulting DSBs are resolved by the HR pathway (104). The NHEJ pathway operates mainly in the G_0_/G_1_ phase of the cell cycle by joining DSB ends, whereas HR employs the sister homolog as the template for repair and thereby is restricted to late S and G_2_ phase (105).

A recent study demonstrated that Pin1 plays an important role in the DNA repair pathway (106, 107). Using a proteomics approach, the authors elegantly isolated a set of novel Pin1 binding proteins, among them a couple of factors known to play a role in DNA repair including MDC1, 53BP1, BRCA1, and CtIP (106). They focused on the Pin1–CtIP link and demonstrated that depletion of Pin1 by RNAi leads to an increased DNA-end resection and decreased NHEJ frequency. In good accordance with these findings, Pin1 overexpression strongly diminished HR frequency. The authors also provided insight in the mechanism by showing that Pin1-depletion leads to aberrant hyper-phosphorylation of the single-strand DNA binding protein RPA2, which serves as a surrogate marker for DNA-end resection (106). The DNA repair protein CtIP is a key regulator of DNA-end resection at DSBs and is essential for the recruitment of additional key factors to the DSBs in S/G_2_ phase (108–110). Interestingly, the authors found that the Ser/Thr–Pro-directed kinase CDK2 phosphorylates CtIP on Thr315 and Ser276 and thereby facilitates complex formation with Pin1, which subsequently mediated isomerization of CtIP. The resulting altered protein conformation impacts on the stability of CtIP and promotes its polyubiquitylation and proteasomal degradation (Figure 2). This regulatory principle facilitates timing of DNA-end resection at break sites during late S/G_2_ phase. Of note, since Pin1 overexpression is frequently observed in cancer, this mechanism is presumably involved in the increased genomic instability observed in human cancer cells. Taken together, Pin1 obviously plays a critical role in coordinating DNA repair pathway choice by suppressing HR and promoting the NHEJ pathway.

## Conclusion and potential future aspects

The identification of Pin1 and the clarification of its underlying enzymology have greatly put forward our knowledge on mechanisms of signal transduction. The intense research activities on Pin1 during the last years generated fascinating novel insight in the function and regulation of this remarkable enzyme and its role in cell signaling and disease. Pin1 provides a highly sophisticated, elegant means to translate pSer/pThr–Pro phosphorylation marks into conformational changes and thus in altered protein function. Since Ser/Thr–Pro phosphorylation is the most abundant post-translational modification in mammalian cells, it appears not very surprising that Pin1 emerged as a central specifier in signal transduction. Despite these facts, however, there is still much to be learned about the biochemistry and biology of Pin1.

For example, numerous Pin1 substrates including p53, HIPK2, and CtIP harbor several pSer/pThr–Pro sites, which critically contribute to Pin1 binding. This raises a number of questions: how is the exact stoichiometry of the Pin1–substrate complexes? Does Pin1 isomerize all pSer/pThr–Pro bonds in its substrates or only a subset? In addition, it is currently unclear whether Pin1 also cross-talks to phosphatases. The timed removal of the pSer/pThr–Pro marks by a given phosphatase would contribute to lock a substrate in a particular conformational state by preventing a backward isomerization reaction.

Even though there is evidence that Pin1 is subject to regulation by post-translational modifications including phosphorylation and SUMOylation, it currently remains unclear whether Pin1 function is also regulated in response to DNA damage. Such regulation might facilitate functional dissection of the DNA damage-associated functions and the cell growth regulatory and mitotic activities.

Beyond its impact on CtIP function, Pin1 has been found to interact with numerous additional factors implicated in DNA repair (106, 107) suggesting a currently unexplored broader function of Pin1 in coordinating DNA repair.

Finally, Pin1 appears to play important roles both in oncogenic and tumor suppressive signaling pathways. To exploit Pin1 function in diseases such as cancer it will be a major effort in the future to dissect the molecular determinants in order to design small molecules for specific interference with its oncogenic and mitotic functions, but to conserve its pro-apoptotic activities, which are necessary for the efficacy of genotoxic stress-inducing cancer therapies.

## Conflict of Interest Statement

The authors declare that the research was conducted in the absence of any commercial or financial relationships that could be construed as a potential conflict of interest.

## Abbreviations

ASPP, apoptosis stimulating protein of p53; ATM, ataxia telangiectasia mutated; ATR, ataxia telangiectasia related; CDK, cyclin-dependent kinase; ChIP, chromatin immunoprecipitation; Chk2, checkpoint kinase 2; DSB, double-strand breaks; DYRK2, dual-specificity regulated kinase 2; HIPK2, homeodomain-interacting protein kinase 2; HR, homologous recombination; iASSP, inhibitory member of the ASPP family; JNK, c-Jun N-terminal kinase; MEF, mouse embryonic fibroblast; NHEJ, Non-homologous end-joining; PPIase, peptidyl–prolyl isomerase; Siah-1, seven *in absentia* homolog 1; TRF1, telomeric repeat binding factor 1.
